# The doctor and patient of tomorrow: exploring the intersection of artificial intelligence, preventive medicine, and ethical challenges in future healthcare

**DOI:** 10.3389/fdgth.2025.1588479

**Published:** 2025-04-03

**Authors:** Paulo Santos, Isabel Nazaré

**Affiliations:** ^1^ Department of Community Medicine, Information and Health Decision Sciences - MEDCIDS, Faculty of Medicine, University of Porto, Porto, Portugal; ^2^ Center for Health Technology and Services Research - CINTESIS@RISE, Faculty of Medicine, University of Porto, Porto, Portugal; ^3^ USF Barão do Corvo, ULS Gaia-Espinho, Vila Nova de Gaia, Portugal

**Keywords:** artificial intelligence, preventive medicine, medical ethics, precision medicine, patient-centred care

## Abstract

Artificial intelligence (AI) 's rapid integration into healthcare transforms medical decision-making, preventive strategies, and patient engagement. AI-driven technologies, including real-time health monitoring and predictive analytics, offer new personalized preventive care possibilities. However, concerns regarding ethical implications, data security, and equitable access remain unresolved. This paper addresses the critical gap in AI integration in preventive healthcare, highlighting statistical evidence of its impact. It also explores the intersection of AI, preventive medicine, and ethical challenges in future healthcare, envisioning the evolving roles of physicians and patients in an AI-integrated ecosystem. A fictional case study projected for 2040, illustrating an entirely digitized, AI-supported healthcare system, frames the discussion about digital health technologies, privacy regulations, and AI's ethical implications in the future of preventive medicine. Digital health interventions powered by AI will facilitate real-time preventive strategies, strengthen patient autonomy, and enhance precision medicine. However, algorithmic bias, data privacy, and healthcare equity challenges must be addressed to ensure AI fosters inclusivity rather than exacerbating disparities. Regulatory frameworks, such as GDPR, provide foundational protections, but further adaptations are required to govern AI's expanding role in medicine. This digital-assisted preventive medicine has the potential to redefine patient-provider interactions, enhance healthcare efficiency, and promote proactive health management. However, achieving this vision requires a multidisciplinary approach involving health professionals, policymakers, and technology developers. Future research should focus on regulatory strategies, digital literacy, and ethical AI implementation to balance innovation with equity, ensuring that digital healthcare remains patient-centered and inclusive.

## 1 Introduction

Artificial intelligence (AI) is reshaping healthcare by redefining the roles of physicians and patients. AI-driven diagnostics, real-time health monitoring, and predictive analytics enable a shift toward preventive, personalized, and data-driven medicine. Advances in wearable technology, big data, and machine learning help anticipate disease risks and tailor interventions. However, ethical dilemmas, algorithmic bias, data security, and the evolving role of healthcare professionals remain significant challenges. Christoph Wilhelm et al. reviewed 19 studies and concluded that AI is still in its early stages and requires further validation before widespread adoption in healthcare (1). Like any technology, AI must be evaluated for its medical, social, economic, and ethical implications (2).

Despite AI's transformative potential, its integration into preventive healthcare remains limited due to regulatory barriers, ethical concerns, and digital literacy disparities. Most AI applications focus on disease treatment rather than prevention, creating a critical gap in proactive healthcare models. Moreover, increasing reliance on automated decision-making raises concerns about privacy, transparency, and the reinforcement of health disparities. A multidisciplinary approach is essential to ensure AI aligns with ethical, human-centered, and equitable healthcare (3).

Healthcare technology now enables procedures once thought impossible, disrupting traditional practices and challenging established routines. Resistance to change often stems from fear of the unknown, leading to inertia. However, digital transformation is inevitable and delayed adoption risks widening the gap between healthcare systems and societal expectations.

This paper explores AI's future role in preventive medicine, envisioning new doctor-patient interactions, digital health ecosystems, and ethical governance. Using a fictional 2040 case study, it illustrates AI-powered healthcare's potential while critically assessing its risks and responsibilities. We hypothesize that while AI-driven preventive medicine can enhance healthcare efficiency and patient autonomy, its widespread adoption faces challenges due to regulatory barriers, ethical concerns, and digital literacy disparities. The discussion underscores the urgent need for responsible AI implementation, regulatory adaptation, and digital health strategies that balance innovation with patient-centered care.

## 2 The future for good

It is 2040.

Mary had just turned 60. She feels good, confident, and functional. She still has a few years of work ahead of her and continues with multiple projects in the team she leads. The kids are grown, and she intends to dedicate more time to social activities. She absentmindedly glances at her smartphone. She checks the emails and WhatsApp messages. A message from the health app catches her attention. Interestingly, the virtual medical assistant hopes she had a great birthday 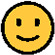 and wishes her lots of health and success for this new year 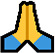. It also suggests to review the health dashboard results 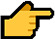.

When she clicks, it opens a set of graphs showing the evolution of the vital parameters over the last few months. She thinks it's great to have the smartwatch and the fitted undershirt. When she was born in the 1980s, she had to go to the health center to schedule an appointment with the doctor.. She looks for the evolution of weight, which has gone up a bit since menopause (all these parties..), and also the body fat mass (Oops! ..), and blood pressure, and blood sugar, and stress levels (which must be from last week's meetings..), and she had been walking less (I'll have to go back to the gym..). There is an increase in overall cardiovascular and oncological risks (is this something to be worried about? ..).

She opens the analysis tab: The probability of a lifetime event now exceeds 40%. It suggests behavior changes, combating the sedentary lifestyle, controlling excess weight, starting medication for blood pressure and cholesterol, carrying out a series of vaccinations, scheduling screening tests for cardiovascular risk, diabetes, breast cancer, of the colon and rectum, stomach and cervix. There is also a depression, stress, and sexuality questionnaire to answer and a genetic test suggestion to personalize the best options for her specific case.

Medicine is more than a biomedical field; it is a social science shaped by emotions, behaviors, and uncertainty. Medical thinking is probabilistic, influenced by multifactorial determinants that resist strict mathematical modeling. Decisions are often dichotomous, yet their impacts unfold in complex, unpredictable ways.

The future remains unwritten, shaped by present choices with irreversible consequences. Even revoked decisions leave lasting effects. History reveals three key insights: first, decisions shape temporal evolution; second, behavioral cycles repeat due to social and cultural influences beyond rational explanation; and third, technological advancements now offer unprecedented precision in health interventions. Concepts like precision medicine, AI, and molecular sciences dominate discussions, reshaping our collective role in healthcare.

**Figure 1:**
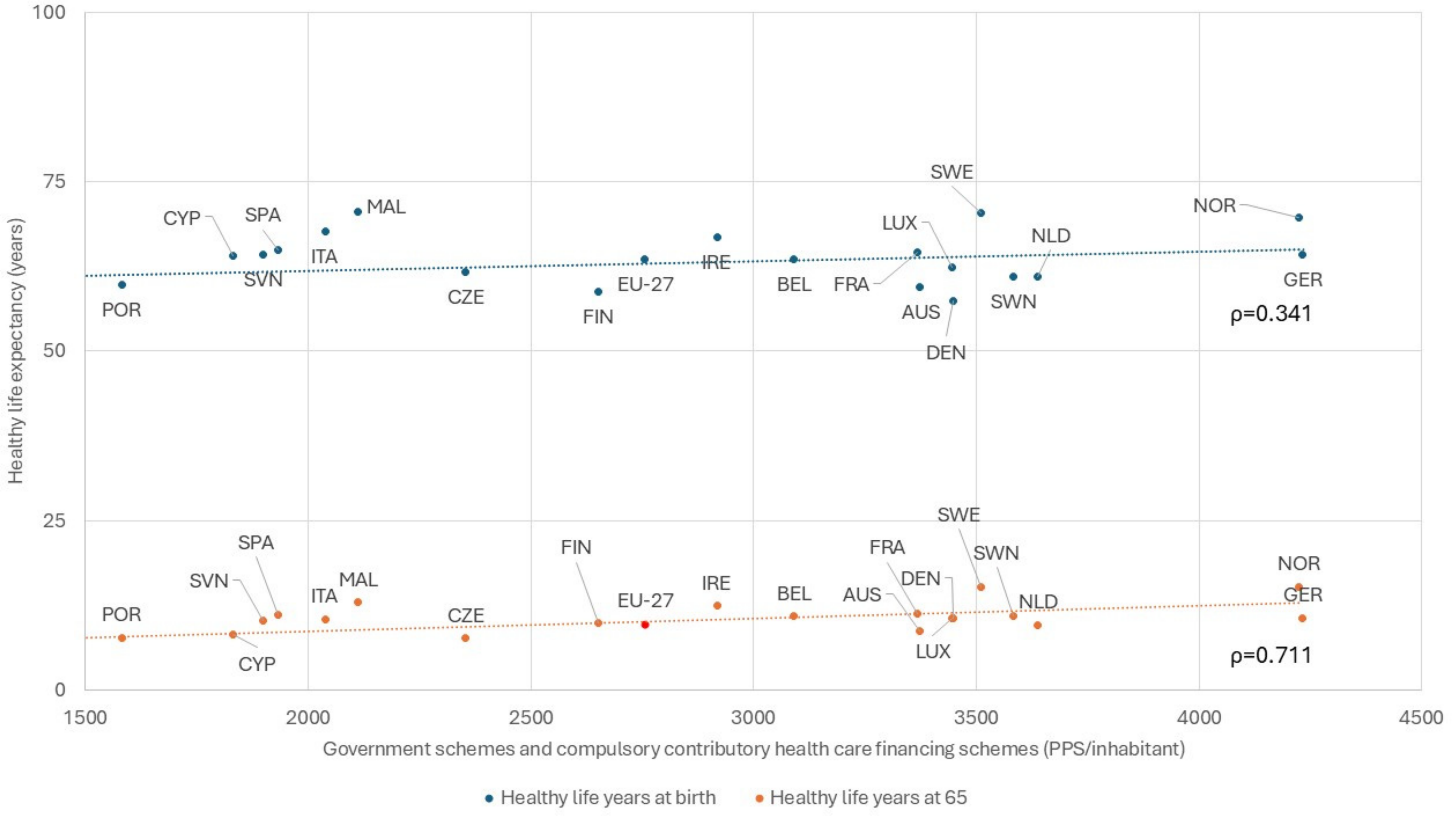
Relation between public expenditure in health care, based on government schemes and compulsory contributory financing schemes and healthy life years across European countries (Eurostat, 2019-22). AUS, Austria; BEL, Belgium; BUL, Bulgaria; CRO, Croatia; CYP, Cyprus; CZE, Czechia; DEN, Denmark; EST, Estonia; EU-27, European Union - 27 countries; FIN, Finland; FRA, France; GER, Germany; GRE, Greece; HUN, Hungary; ICE, Iceland; IRE, Ireland; ITA, Italy; LVA, Latvia; LTU, Lithuania; LUX, Luxembourg; MAL, Malta; NLD, Netherlands; NOR, Norway; POL, Poland; POR, Portugal; ROM, Romania; SVK, Slovakia; SVN, Slovenia; SPA, Spain; SWE, Sweden.

Europe's landmark decision to establish a social healthcare system, ensuring equitable access regardless of funding models, has contributed to better health outcomes at lower costs than the United States (4). Viewing health as a public investment has significantly extended longevity, particularly for individuals over 65 (Figure 1). This foundational decision continues to influence public health strategies and will shape the future of medicine.

**Figure 2:**
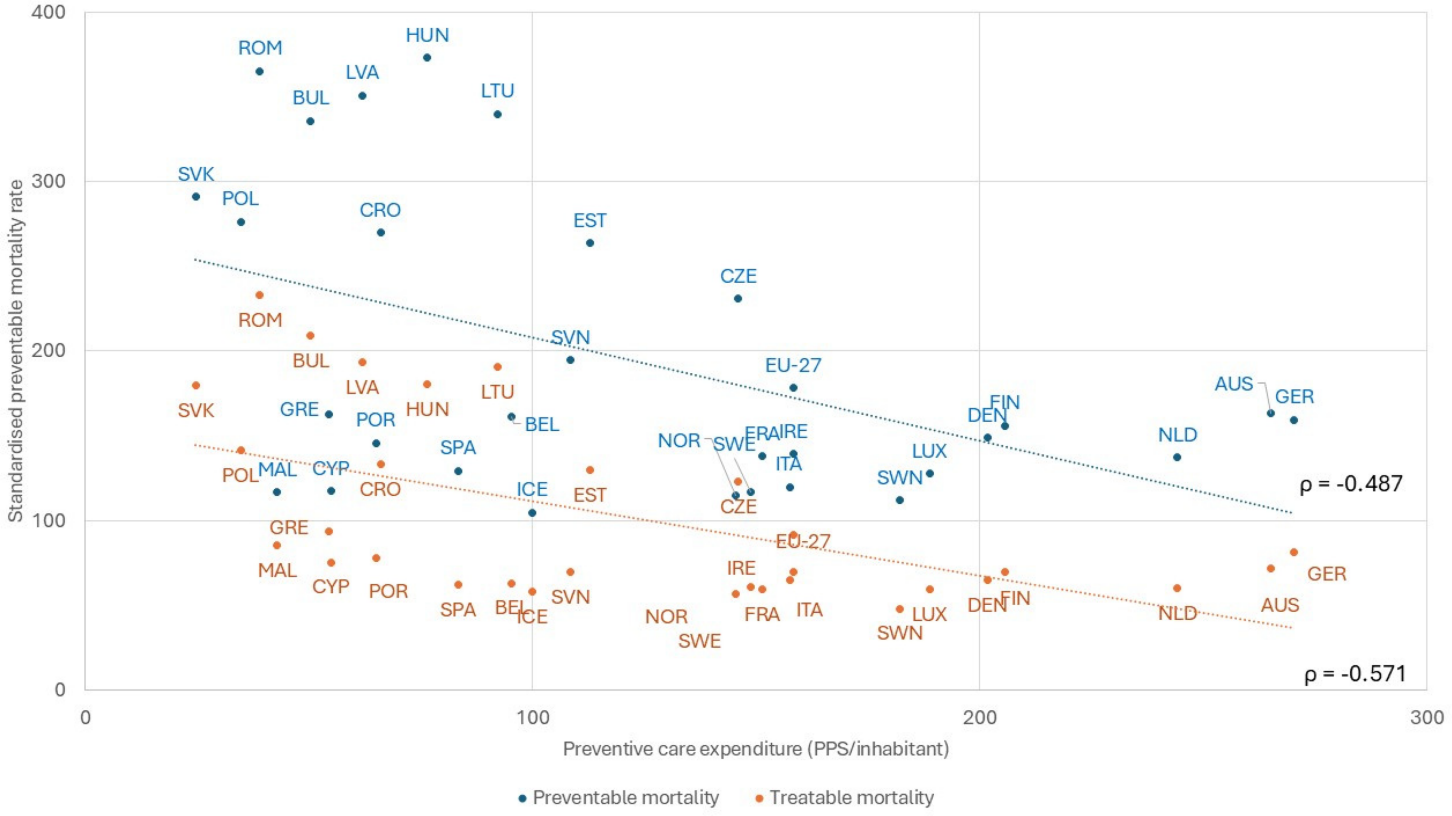
Relation between investment in primary care, measured by preventive care expenditure, and preventable mortality (Eurostat, 2019-21). AUS, Austria; BEL, Belgium; BUL, Bulgaria; CRO, Croatia; CYP, Cyprus; CZE, Czechia; DEN, Denmark; EST, Estonia; EU-27, European Union - 27 countries; FIN, Finland; FRA, France; GER, Germany; GRE, Greece; HUN, Hungary; ICE, Iceland; IRE, Ireland; ITA, Italy; LVA, Latvia; LTU, Lithuania; LUX, Luxembourg; MAL, Malta; NLD, Netherlands; NOR, Norway; POL, Poland; POR, Portugal; ROM, Romania; SVK, Slovakia; SVN, Slovenia; SPA, Spain; SWE, Sweden.

Personalizing evidence in practical healthcare relies on patient-centered systems. While technology focuses on disease treatment, its impact depends on addressing patient needs, benefits, and concerns. Strong primary care enhances accessibility and adherence to good clinical practices, ensuring effective integration of medical advancements into everyday health management (Figure 2).

Moreover, technological acceleration through digitalization enhances precision diagnostics and preventive care. Advanced algorithms analyze vast data, revealing predictive insights for early intervention (5, 6). Salinas et al. recently systematically reviewed fifty-three studies comparing the performance of AI algorithms for skin cancer classification to clinicians. She found an overall sensitivity of 87.0% and specificity of 77.1% for AI algorithms and 79.8%/73.6% for all clinicians (*p* < 0.001), which also remained the same in comparison with highly trained dermatologists (7). Wearable health devices enable real-time patient tracking, offering immediate feedback to guide health behaviors (8). However, information alone does not drive change—activation is crucial for action (9, 10).

**Figure 3:**
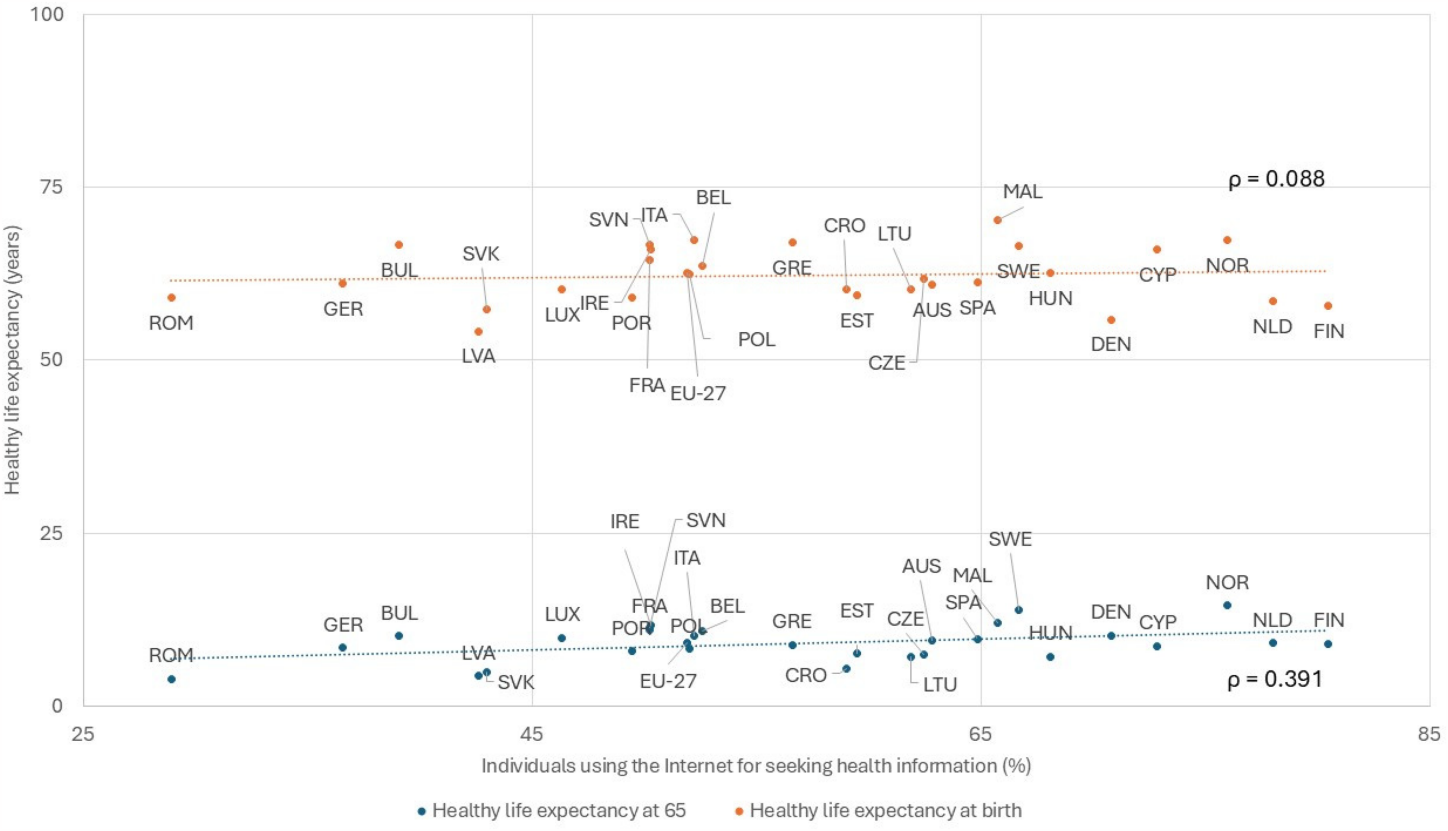
Relation between the proportion of individuals seeking health information on the internet in the last 3 months and healthy life expectancy (Eurostat, 2019-22). AUS, Austria; BEL, Belgium; BUL, Bulgaria; CRO, Croatia; CYP, Cyprus; CZE, Czechia; DEN, Denmark; EST, Estonia; EU-27, European Union - 27 countries; FIN, Finland; FRA, France; GER, Germany; GRE, Greece; HUN, Hungary; ICE, Iceland; IRE, Ireland; ITA, Italy; LVA, Latvia; LTU, Lithuania; LUX, Luxembourg; MAL, Malta; NLD, Netherlands; NOR, Norway; POL, Poland; POR, Portugal; ROM, Romania; SVK, Slovakia; SVN, Slovenia; SPA, Spain; SWE, Sweden.

Despite growing digital maturity in Europe, healthcare lags behind industries like finance and telecommunications. While patients increasingly use digital tools for health management, true literacy goes beyond access to information. Notably, there is a weak correlation between online health searches and improved health outcomes, highlighting the need for more effective digital health strategies (Figure 3).

Maria thinks it is easy to find her routes and health options. The always-accessible app made available information in the comfort of her smartphone. Continuous feedback from her health monitoring allows her to take appropriate action in real time. “What a difference from my younger days!” It improved accessibility, the opportunity for intervention, and the intelligibility of the available information. However, Maria also thinks about the security of this data: who can access it? The employer? the hackers? The insurance company? The State? What implications will this have? “Perhaps it is better to wait before making the genetic test … at least for now.”

## 3 Privacy and security considerations

Despite the promise of digital healthcare, data security remains a major concern. Reports of data abuse, misuse, and overuse highlight vulnerabilities in digital systems, often underestimated until individuals experience them firsthand. The Internet of Things and social media enable inferences about personal attributes, from intelligence to health risks, raising privacy concerns (11).

Many patients unknowingly share data online without understanding privacy policies or future uses (12). While legal frameworks exist, individuals often consent to terms without reading them. Ethical responsibility extends beyond formal authorization—companies must minimize harm, distribute benefits fairly, and respect autonomy (13). Genuine autonomy builds through ongoing interactions between doctors and patients (14), a relationship not fully replicated by digital tools due to risks like hacking and spyware.

For AI-driven healthcare to be trustworthy, transparency, accountability, and inclusivity are essential (13). AI's statistical nature risks reinforcing biases, favoring common cases over rare conditions (15). Governments and regulators, such as through GDPR (15), play a crucial role in enforcing encryption, anonymization, and data access rights, ensuring individuals retain control over their health information (16).

## 4 Ethical weighing

For Maria, this application brings indisputable advantages and trust in the regulatory framework that keeps her data safe and inviolable. Moreover, she is committed to contributing to overall health improvement, giving their data to the big bag, which will be gathered with large volumes of collective data and analyzed for the benefit of all.

Periodically, the virtual assistant suggests small, determined goals to Maria, highlighting how each step can improve her quantity and quality of life. This facilitates motivation and confidence to implement the proposed changes. Maria is just one example of how technology can transform the individual health experience, with an impact far beyond the individual, influencing entire systems and communities.

The future doctors will vastly differ from today's, just as modern medicine has evolved from its historical roots. Initially focused on curing diseases, medicine later embraced prevention, formalized by the Ottawa Charter, emphasizing health promotion and patient education. However, behavioral change remains challenging, especially in primordial prevention, where healthy individuals often lack the motivation to adopt healthier lifestyles. Overcoming this requires interventions outside clinical settings, leveraging technology such as patient engagement platforms and AI-driven decision support (17).

Future doctors will be medical experts and, at the same time, technology and data science specialists. They will integrate clinical practice with advanced analytics, bioinformatics, and engineering, leading multidisciplinary teams across healthcare, education, security, and urban planning. This shift will enable a proactive, data-driven approach to healthcare, using AI-generated algorithms to anticipate health risks and provide real-time, personalized interventions.

Medicine will become increasingly holistic, integrating molecular-level insights from precision medicine and “omics” sciences to tailor preventive and therapeutic strategies (18). Ethical considerations will be central, with beneficence becoming more precise and autonomy strengthened through informed patient-provider relationships. However, equity will remain a challenge, as personalized medicine may be costly despite technological advancements potentially lowering prices. The key lies in prioritizing those most in need and ensuring that AI-driven healthcare serves society rather than exacerbating disparities.

Physicians will continue innovating and adapting scientific knowledge to individual cases while addressing emerging challenges. However, the bureaucratic control of medicine has historically stifled creativity, leading to identity crises among doctors who feel disconnected from their mission (4). Despite technological progress, medicine will always be rooted in the human relationship between caregiver and patient.

The doctor-patient relationship will evolve into a hybrid model where AI enhances decision-making, but human oversight remains crucial for trust and empathy (19). Physicians will bridge the gap between technological innovation and equitable care, ensuring that AI is not a barrier but a tool for improving health outcomes (1). Digital literacy and accessible technologies will be essential in reducing inequalities and empowering individuals to actively participate in their healthcare while maintaining the foundational humanistic values of medicine (20).

## 5 The doctors of tomorrow

Future doctors will integrate seamlessly into people's lives through digital platforms, offering in-person and remote care while respecting autonomy and free will. They will act as custodians of vast health data, ensuring confidentiality and leading multidisciplinary teams that provide comprehensive, patient-centered care.

Despite technological advancements, their fundamental role remains unchanged—offering relief, guidance, and support, as Voltaire once described. Medicine's evolution must balance innovation with its founding ethical principles, prioritizing patient expectations, values, and human connection. Physicians are not just healers but individuals with aspirations and responsibilities, shaping the future of healthcare with innovation, empathy, and ethical commitment.

Evidence-based medicine must continue to integrate ethical and humanistic foundations with scientific precision. The doctor-patient relationship remains central, ensuring that technology enhances, rather than replaces, the essence of medical care.

## 6 Conclusion

AI integration in preventive medicine shifts healthcare from reactive to proactive and personalized care. Digital tools, including real-time health monitoring and predictive analytics, improve early disease detection and empower individuals in health management. However, successful AI implementation depends on strong privacy, security, and fairness standards to prevent biases and protect patient data.

Regulatory frameworks must evolve to ensure AI transparency, accountability, and equitable access. Despite AI's promise, limitations include algorithmic biases, data privacy concerns, and the digital divide, which may exclude vulnerable populations. Future studies should explore mitigation strategies, such as adaptive AI models and improved data security protocols. The leadership of multidisciplinary teams should remain with physicians, who must balance technological efficiency with human empathy to keep healthcare patient-centered.

The future of AI in medicine requires collaboration between healthcare providers, data scientists, and policymakers, ensuring ethical deployment and enhancing digital literacy. Future research should focus on integrating AI into primary care, refining regulatory models, and ensuring AI-driven healthcare remains inclusive.

Ultimately, medicine is not just about technology but a human-centered transformation. Ensuring AI supports patient-centered care demands a steadfast commitment to equity, privacy, and ethical governance. Future research should prioritize responsible AI deployment, accessibility, and regulatory frameworks that promote fairness and transparency across global healthcare systems.

## Data Availability

The raw data supporting the conclusions of this article will be made available by the authors, without undue reservation.
